# Editorial: System biology to regulatory grids: new tools and clues aimed at improving plant evolutionary-developmental (Evo-Devo) biology

**DOI:** 10.3389/fpls.2024.1380569

**Published:** 2024-03-18

**Authors:** Elisson Romanel, Henrique Cestari DePaoli, Michael dos Santos Brito

**Affiliations:** ^1^ Lorena School of Engineering, University of São Paulo, Lorena, Brazil; ^2^ BSE Division, Biodesign Department, Lawrence Berkeley National Laboratory, Berkeley, CA, United States; ^3^ Institute of Science and Technology, Federal University of São Paulo, São José dos Campos, Brazil

**Keywords:** system biology analysis, plant biotechnology, plant evolutionary-developmental (Evo-Devo), plant genetics and genomics, transgenic plants, regulatory grids

The intricate choreography between genetics and environmental factors shape the development of plants and have always been a subject of fascination for scientists and researchers. The field of plant evolutionary-developmental biology (Evo-Devo) continues to unfold, and the integration of systems biology and regulatory grids emerges as a promising avenue for gaining deeper insights into the known and unknown mechanisms governing plant growth and adaptation in different scenarios. In this Research Topic, we look forward to the significance of this intersection, exploring the potential of new clues and tools that deliver results for a better understanding of plant systems biology. By adopting a systems-level view, researchers gain a more comprehensive understanding of how molecular components collaborate to orchestrate the intricate processes of plant development. In this context, regulatory grids, serve as the central nexus in understanding the regulatory networks governing plant Evo-Devo, at different levels and node connections. These grids encompass the myriad of genetic and epigenetic elements that participate into the fate of cells, tissues, and organs throughout a plant’s life cycle. Untangle nodes of these regulatory grids pave new avenues for manipulating plant traits and enhancing agricultural productivity.

Complementary to this, the understanding of plant Evo-Devo harnesses this knowledge not just for the improvement of crops, but also to understand conserved and divergent plant molecular processes. By identifying key regulators and deciphering the intricate signaling pathways, researchers can develop strategies to enhance traits such as yield, water-use efficiency, and nutritional content. The potential for targeted crop improvement holds promises for addressing the challenges posed by a growing global population and mitigation of the greenhouse effect. Both exploratory and fact-based work within the aforementioned points were carefully selected here and represent a small yet diverse set of information for Evo-Devo biology:

Jiang et al. explore a fundamental question in plant evolution regarding the origin of seeds and the development of integument structures. The study investigates whether integument arises *de novo* or evolves from pre-existing structures by examining the key regulatory genes involved in integument development. Through a comprehensive analysis of the origin and evolution of genes such as ANT, CUC, BEL1, SPL, C3HDZ, INO, ATS, and ETT in seedless plant genomes, the authors identify duplication-divergence events and motif changes in coding regions. Expression and functional studies reveal that these genes play crucial roles in the genetic program shaping leaf-like lateral organs, suggesting serial homology between integuments and other lateral structures. The manuscript proposes a successive transformation model for integument origins, arguing against *de novo* emergence and instead supporting evolution from pre-existing, serially homologous structures, although the precise signal or trigger for this transformation remains unknown. Since seeds represent the primary source of stem cells for plant development, the genetic network of this work contributes valuable insights into the molecular evolution and developmental processes underlying specialized tissue emergence and its correlation with seedless plant organ/tissue development. In the manuscript titled “*The root signals in rhizospheric interorganismal communications*” authors describe the role of root exudates in mediating complex interactions within the rhizospheric environment. Root exudates, comprising various metabolites, are conductors of plant-plant and plant-rhizomicrobiome communications. By influencing the biochemical and physiological aspects of associated microorganisms, these signals contribute to enhanced plant growth and resilience. The review highlights the dynamic nature of root exudate production and emphasizes the need for a deeper understanding of the metabolites and metabolic pathways involved in inter-organismal communications (Lyu and Smith). Such complexity of plant-microbe interaction is analogous to events coordinating sexual plant reproduction, and both are ‘rooted with the environment’ (Angulo et al., 2022). Not surprisingly, the latter network chronologically derives from flower development, where the exploration of flowering transition pathways and determination of gene regulatory networks unfolds a dynamic system-level model for the flowering process in Arabidopsis thaliana (Chávez-Hernández et al.). Such Flowering Transition Gene Regulatory Network (FT-GRN), included novel experimental data on the MADS-box transcription factor XAANTAL2 (XAL2), which coordinates stem-cell patterning as well as root and SAM meristem proliferation, and serves as a robust biological module, offering a dynamic systems biology mechanism that integrates genetic flowering pathways to elucidate SAM phase transitions, pre-reproduction.

Another work reanalyzes available data to propose a paradigm shift in conceptualizing the informational ecosystem guiding plant tissue and organ growth. The author disputes the thoroughgoing of current theoretical frameworks and suggests an alternative perspective that incorporates the stochastic nature of molecular signaling (Lintilhac, 2022). Still in this direction, Kumar et al. investigate the regulatory role of N-Acetylserotonin O-methyltransferase (ASMT), the final enzyme in melatonin biosynthesis, in soybean development and its correlation with soybean under stress conditions. Such category of enzymes are well known to have a broad set of functions in- and out- of the photosynthetic world (Murch and Erland, 2021). Nevertheless, through a comprehensive evo-devo-linked analysis, which includes genome-wide examination, gene structure analysis, cis-acting elements identification, and enzymatic assays, the study identifies 44 GmASMTs in soybean with traditional and segmental duplications. Co-expression networking highlights the involvement of distinct GmASMTs in various stress responses, such as embryo development, heat, drought, aphid, and soybean nematode infections, forming associations with key elements in stress regulation, including some classical transcription factors (NAC, MYB, WRKY, and ERF), stress signaling, isoflavones, secondary metabolites, calcium, and calmodulin proteins. Moreover, GmASMTs exhibit auxin-like activities, influencing genes related to auxin transport and auxin-responsive proteins during plant developmental programs and when faced with different types of stress. Finally, in the article “*Glucose Supply Induces PsMYB2-Mediated Anthocyanin Accumulation in Paeonia suffruticosa ‘Tai Yang’ Cut Flower*”, the author addresses the color fading issue in cut flowers of tree peony, a popular Chinese ornamental plant (Zhang et al.). This study reveals that exogenous glucose supply significantly enhances the color quality of P. suffruticosa ‘Tai Yang’ cut flowers by increasing total soluble sugar and anthocyanin contents in petals. The research identifies PsMYB2 as a key regulatory gene using transgenic plants approach, correlating PsMYB2 with upregulation of anthocyanin biosynthetic genes. This research largely contributes to our understanding of the molecular basis for color enhancement in tree peony cut flowers and lays the groundwork for the development of color retention technologies in the cut flower industry.

According to the different papers but also with different tools and issues described, it is clear the integration of systems biology and regulatory grids offers significant potential toward a global comprehension of plant development. Therefore, the convergence of systems biology and regulatory grids marks a pivotal moment in the study of plant Evo-Devo. This Research Topic advocates for a collaborative and interdisciplinary approach, bringing together researchers from diverse fields to unravel the mysteries of plant development using discovery-, in silico-, and hypothesis-driven approaches. By embracing alternative perspectives and unconventional tools, clues bloom, understanding zoom, innovations spume, and global challenges in agriculture and food security will not gloom (Rangan et al., 2023). The journey ahead is challenging, but the potential for transformative discoveries makes it a venture well worth undertaking, and perhaps radiant.

## Author contributions

MSB: Writing – original draft, Writing – review & editing. ER: Writing – original draft, Writing – review & editing. HD: Writing – original draft, Writing – review & editing.
